# Murine Typhus in Drug Detoxification Facility, Yunnan Province, China, 2010

**DOI:** 10.3201/eid1808.120060

**Published:** 2012-08

**Authors:** Wei-Hong Yang, Tuo Dong, Hai-Lin Zhang, Shi-Wen Wang, Hui-Lan Yu, Yu-Zhen Zhang, Yong-Hua Liu, Zheng-Liu Yin, Yun Feng, Zhang-Yi Qu, Jian-Guo Xu, Li-Juan Zhang

**Affiliations:** Yunnan Institute of Endemic Diseases Control and Prevention, Dali, People’s Republic of China (W.-H. Yang, H.-L. Zhang, Y.-Z. Zhang, Y. Feng);; People’s Republic of China ICDC, Beijing, People’s Republic of China (T. Dong, S.-W. Wang, H.-L. Yu, J.-G. Xu, L.-J. Zhang);; Harbin Medical University, Harbin, People’s Republic of China (T. Dong, Z.-Y Qu);; and Ruili Center for Disease Control and Prevention, Ruili, People’s Republic of China (Y.-H. Liu, Z.-L. Yin)

**Keywords:** Murine typhus, Rickettsia typhi, outbreak, China–Myanmar border, People’s Republic of China, typhus, Rickettsia, vector-borne infections

**To the Editor:** An outbreak of murine typhus caused by *Rickettsia typhi* was confirmed among persons attending a 51-acre drug detoxification program 2.5 km from Ruili City in Yunnan Province, People’s Republic of China. Ruili City, with an average altitude of 1,381 km, is located in southwestern China near the Myanmar border (Figure). At the time of the outbreak, the detoxification program had 1,264 inpatients and 96 staff members. The facility is divided into sections A (women), B, C, and D. Residents of each section are housed in a 4-story building; each floor contains 9 rooms (2 m^2^ per person). During September 4–21, 2010, a total of 76 of the 430 residents of section B were reported with fever of unknown cause. All patients were men 19–38 years of age who worked in clothing manufacture at the facility and were receiving treatment for drug addiction. Before the outbreak, rats and stray cats were frequently observed in a cafeteria in section B. No persons with similar illness were observed in the other 3 sections.

**Figure:**
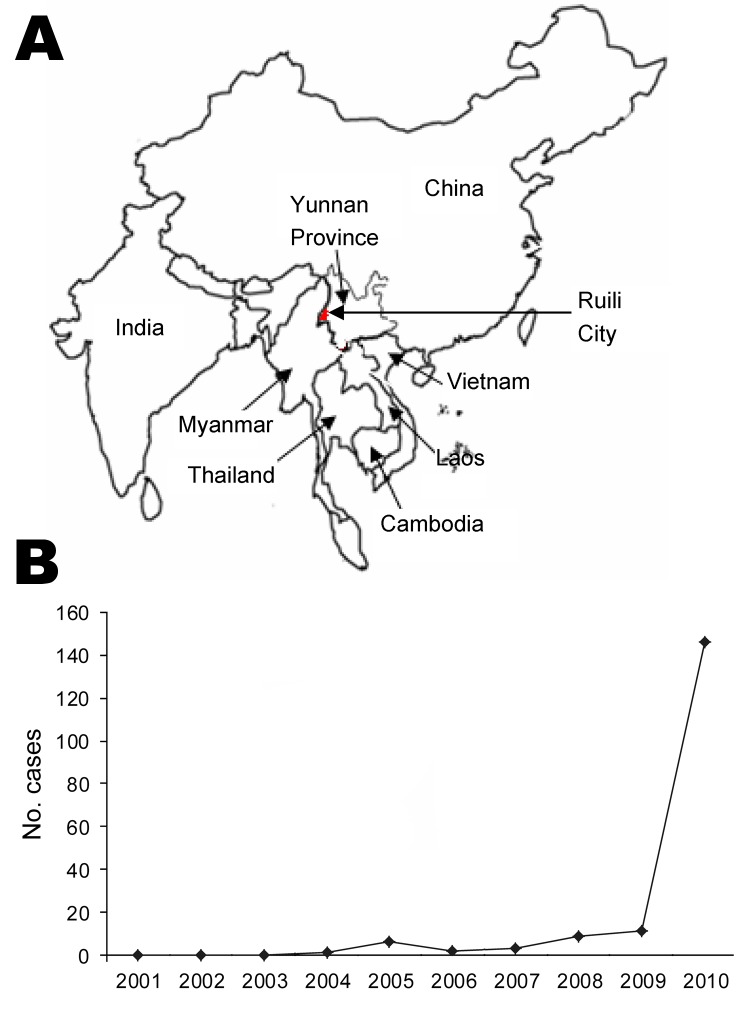
A) Location of Ruili City, Yunnan Province, People’s Republic of China (97°51′–98°02′E, 23°38′–24°14′S; altitude 1,381 m). B) Number of murine typhus cases reported from Ruili City Center for Disease Control and Prevention during 2001–2010.

To investigate the outbreak, we gathered information about demographics; past medical histories; exposures to vectors, such as ticks, mites, fleas, and lice; and symptoms. Patients frequently reported headache, dizziness, diffuse myalgia, high fever (>39°C), and shivers but did not report a rash or eschar. No patients remembered a flea or louse bite, but they frequently reported seeing rats in the area. The Chinese Center for Disease Control and Prevention (China CDC) Institutional Review Board approved the investigation.

Two milliliters of blood was collected from each consenting patient. Separated serum and the remaining blood clots were stored at −70°C and transferred to the Department of Rickettsiology, National Institute of Communicable Disease Control and Prevention, China CDC, for testing. Specimens were tested by indirect immunofluorescence assay (*1*) to detect specific IgM and IgG against 10 common rickettsiae: *Rickettsia prowazekii*, *R. typhi*, *R. heilongjiangensis*, *Orientia tsutsugamushi* types Karp and Kato, *Coxiella burnetii*, *Bartonella henselae*, and *B. quintana*, *Ehrlichia chaffeensis*, and *Anaplasma phagocytophilum*. Antigens were prepared by placing the rickettsial stains in L929 cells and HL60 or and DH82 cells, respectively; collecting the culture when Gimenez stain or Wright staining showed positive results; ultrasonically crushing the culture; and purifying the bacteria by density ultracentrifugation. Positive control serum was prepared by inoculating rabbits with the above standard rickettsiae strains.

We collected 76 serum samples from patients a median of 4 days (range 1–9 days) after illness onset. Thirty-five (40%) were IgM positive for *R. typhi* (titer >40, maximum titer 160) and 29 (38%) were IgG positive for *R. typhi* (titer >80, maximum titer 320). No samples were positive for the other 8 rickettsial antigens, except for 10 (13%) that had weak reactions for *R. prowazakii* (titer 40). Twelve convalescent-phase serum samples (median interval between acute and convalescent phases 187 days [range 181–192 days]) were IgG positive for *R. typhi* (titer >80) and 4 had 4-fold increases in titer; 2 reached titers of 1,280 and 2,560.

DNA was extracted from acute-phase samples by using a QIAGEN DNA extraction kit (Hilden, Germany) and tested by real-time PCR that targeted the *groEL* gene *of R. prowazekii* and *R. typhi* (*2*). Twelve (16%) of the 76 samples were positive. To differentiate between *R. prowazekii* and *R. typhi*, we used a previously developed nested PCR targeting the *groEL* gene *of R. prowazekii* and *R. typhi* (*3*) and found the expected 218-bp fragments in 11 patients. BLAST analysis (http://blast.ncbi.nlm.nih.gov/Blast.cgi) showed that these sequences (200 bp) were 100% homologous with that of *R. typhi* strain Wilmington (GenBank accession no. AF017197).

Initially, patients were treated with antiviral drugs and Chinese herbal medicine for suspected influenza. Subsequently, murine typhus was suspected and doxycycline was administered. All patients recovered fully.

Yunnan Province’s subtropical geographic and climate characteristics are advantageous to the vectors of rickettsial diseases, such as murine typhus, scrub typhus, spotted fever, and Q fever (*4**–**6*). Three national murine typhus outbreaks involving >10,000 cases each have been reported since 1949, and each involved Yunnan Province (*7*). In the 1970s, an outbreak of louse-borne typhus occurred in northeastern Yunnan Province (*4*); since then, louse-borne typhus has been rarely reported. Murine typhus was reported from Baoshan City, east of Ruili City, in 2010. However, the currently reported murine typhus outbreak in Ruili City near the China–Myanmar border was the largest outbreak in China during the previous decade. None of the 76 patients had rash, a finding similar to that reported in previous outbreaks in Myanmar, Thailand, and other Southeast Asia regions (*8**–**10*). In addition to the 76 cases reported here, 70 additional sporadic cases of murine typhus were reported to the Ruili CDC in 2010. We conclude that murine typhus should be considered in cases of unexplained fever with nonspecific clinical manifestations in southern Yunnan Province.
